# Heart Morphometry in Standard Second Trimester Scan

**DOI:** 10.3390/diagnostics15233088

**Published:** 2025-12-04

**Authors:** Alexandru-Cristian Comănescu, Dragoș-Ovidiu Alexandru, Maria-Cristina Comănescu, Agnesa Preda, Mar Bennasar

**Affiliations:** 1 Department of Obstetrics and Gynaecology, University of Medicine and Pharmacy Craiova, 200349 Craiova, Romania; alexcom8000@yahoo.com; 2 Department of Medical Informatics and Statistics, University of Medicine and Pharmacy Craiova, 200349 Craiova, Romania; 3 Department of Anatomy, University of Medicine and Pharmacy Craiova, 200349 Craiova, Romania; 4 Department of Obstetrics and Gynaecology, Emergency County Hospital Craiova, 210218 Craiova, Romania; 5 BCNatal–Fetal Medicine Research Center, Hospital Clınic and Hospital Sant Joan de Deu, University of Barcelona, 08007 Barcelona, Spain

**Keywords:** anomaly scan, fetal heart, cardiac morphometry

## Abstract

**Introduction:** Routine second trimester anomaly scans include standard cardiac planes, yet detailed cardiac morphometry is not part of current practice. We hypothesized that a comprehensive set of cardiac measurements could be obtained from these standard views without prolonging examination time and with clinically meaningful reproducibility. **Methods:** We conducted a prospective study involving ninety-two uncomplicated singleton pregnancies undergoing routine second trimester anomaly scans. Cardiac measurements were obtained using standard ISUOG/SRUOG planes, both during the examination and offline. Feasibility, reproducibility, and the impact on scanning time were evaluated, and results were compared with established reference ranges. **Results:** All morphometric measurements were successfully obtained in 100% of included cases. Mean “screen time” increased only minimally from 35.45 min (95% CI 32.9–38.0) to 38.75 min (95% CI 36.1–41.4), with a non-significant mean difference of 3.30 min (*p* = 0.063). Most z-scores fell within ±2 SD. Intra-observer reproducibility ranged from fair to excellent, with strong correlations for major cardiac dimensions (r > 0.80 for multiple parameters). **Conclusions:** Comprehensive fetal cardiac morphometry can be integrated into the routine second trimester anomaly scan using standard imaging planes, without prolonging the examination. This approach may support earlier recognition of atypical growth patterns or cardiac remodeling.

## 1. Introduction

### 1.1. Theoretical Framework

#### 1.1.1. Fetal Heart Evaluation in Standard Second Trimester Scan

**Figure 1:**
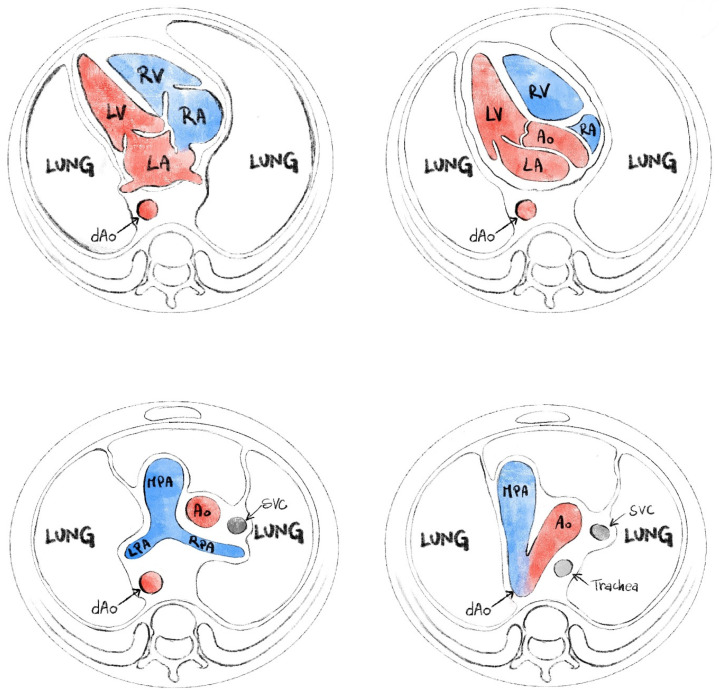
Standard planes for evaluation of the fetal heart (from ISUOG fetal heart screening—modified).

The routine second trimester scan includes an evaluation of the fetal heart on standard planes (Figure 1). The four-chamber view is expected to offer us information about situs and general appearance, atrial and ventricular size, and atrioventricular valves function. Right and left ventricular outflow tracts visualization is required, with subjective appreciation of their size. The left ventricular outflow tract (LVOT) should be shown exiting from the left ventricle with aorta-septal continuity. RVOT (right ventricular outflow tract) should be seen rising from the right ventricle and bifurcating. The three vessels view and three vessels trachea view are part of the examination and can help detect anomalies that involve the outflow tracts or veins (e.g., transposition of great arteries, persistent left superior vena cava, aortic coarctation). These planes are standard in most international and national guidelines [1,2,3,4,5] and are generally required as standard images ideally accompanied by short clips (showing the movement of the atrioventricular valves or the great vessels crossing-over).

#### 1.1.2. Fetal Cardiac Morphometry

Cardiac morphometry in fetuses during the second trimester involves the measurement and assessment of various cardiac dimensions and geometric parameters using echocardiography. This includes evaluating the relative size of the heart, ventricles, and atria, as well as their sphericity, ventricular dominance, wall asymmetry, and relative wall thickness.

A study by García-Otero et al. constructed nomograms for these parameters from 18 to 41 weeks of gestation, demonstrating that these measurements are feasible and reproducible in over 95% of cases. Key parameters assessed included the cardiothoracic ratio, atrial-to-heart area ratios, ventricular-to-heart area ratios, and sphericity indices. The study found that while some parameters like the cardiothoracic ratio and ventricular right-to-left ratio modestly increase throughout gestation, others, such as atrial sphericity indices and septal-to-free wall thickness ratios, remain constant [6]. Additionally, Frandsen et al. highlighted that growth-restricted fetuses exhibit smaller cardiac dimensions, including the diameters of the aortic and pulmonary valves, and smaller ventricular sizes, even after adjusting for abdominal circumference [7]. This suggests that intrauterine growth restriction is associated with altered cardiac growth early in pregnancy. These findings help the understanding of normal and pathological cardiac development in fetuses, aiding in the early diagnosis, and management of congenital heart diseases and growth restrictions.

Studies have demonstrated that fetal cardiac measurements can be reliably obtained during the second trimester using advanced ultrasound techniques. For instance, the study by Firpo et al. showed that fetal cardiac dimensions, including ventricular and atrial cavity dimensions, ventricular wall thickness, and valve annulus dimensions, can be measured reliably from 16 weeks of gestation onward using a transabdominal approach [8].

Cardiac morphometry in fetuses during the second trimester can exhibit variations across different populations due to genetic, environmental, and methodological factors. A study by Lussier et al. [9] established reference ranges and z-scores for fetal cardiac measurements specifically in an East Asian cohorts population, highlighting that these measurements can differ from those derived from North American and European populations. This study involved 575 healthy pregnant Taiwanese women and provided percentile graphs for thirteen fetal echocardiographic measurements. This study supports the importance of population-specific reference ranges and the potential for significant differences in fetal cardiac morphometry across various populations. This variability requires the use of tailored reference standards to accurately assess and manage fetal cardiac development in diverse populations.

Another aspect of cardiac morphometry is observed in the assessment of cardiac remodeling. Although no formal consensus exists, fetal cardiac remodeling patterns are described for a large range of pathologies by assessing cardiac, ventricular, and atrial areas, ventricular sphericity indices, septal wall thickness, and relative wall thickness, all adjusted for gestational age or estimated fetal weight [10]. Various techniques for heart measurements can be found in the studies of Garcia-Otero, Schneider, Pasquini, or Vigneswaran [6,11,12,13]. Fetal cardiac morphometry is not routinely performed during a standard second trimester anatomy scan. The primary purpose of the routine second trimester ultrasound, typically performed between 18 and 22 weeks of gestation, is to assess fetal anatomy and identify any structural anomalies. This includes a basic evaluation of the fetal heart, focusing on the four-chamber view and outflow tracts to screen for major congenital heart defects [14,15,16]. However, detailed fetal cardiac morphometry, which involves precise measurements of cardiac structures, is generally reserved for cases where there is a specific indication or increased risk of congenital heart disease. Such indications may include abnormal findings on the initial scan, a family history of congenital heart disease, or other risk factors such as increased nuchal translucency [17]. The American Society of Echocardiography recommends that detailed fetal echocardiography, which includes comprehensive cardiac morphometry, be performed in high-risk pregnancies or when abnormalities are detected during the routine scan [14].

In recent years, automatic and semi-automatic cardiac morphometry tools have gained increasing attention, particularly systems such as FetalHQ, which enable speckle-tracking-based assessment of ventricular geometry and deformation. Although these approaches hold promise for standardizing measurements and reducing operator dependency, their clinical integration remains limited by technical challenges, image-quality requirements, and variability in acquisition protocols. Recent evaluations, including latest version of FetalHQ [18], highlight both the potential of these methods and their current constraints. For this reason, manual measurements remain highly relevant, especially in routine second trimester anomaly scans where feasibility and reproducibility must be ensured even without advanced software.

Although previous studies have established normative ranges for multiple fetal cardiac parameters, only a few have explored whether a comprehensive morphometric assessment can be realistically incorporated into the routine second trimester anomaly scan. The present study contributes by evaluating the feasibility, reproducibility, and time impact of integrating a broad set of cardiac measurements into standard screening images in a real-world clinical setting.

### 1.2. Hypothesis and Objectives

Our hypothesis was that it is possible to perform a cardiac morphometric evaluation during the routine second trimester scan.

The general objective was to perform a comprehensive morphometric cardiac evaluation in routine second trimester anomaly scan—using the standard views that are mandatory for this scan. Specific objectives were meant to cover the following five points:

1. Feasibility: to prove the measurements can be made every time.
2. Measurement analysis: to evaluate our results and compare them with other studies with the same type of population and methodology.
3. Time: to analyze how much time this adds to the usual ultrasound scan.
4. Reproducibility: to analyze if there is an intra-observatory difference.
5. Learning curve: to verify if measurements’ quality (assessed by z-score deviation) improved in the second part of the project.

## 2. Materials and Methods

**Figure 2:**
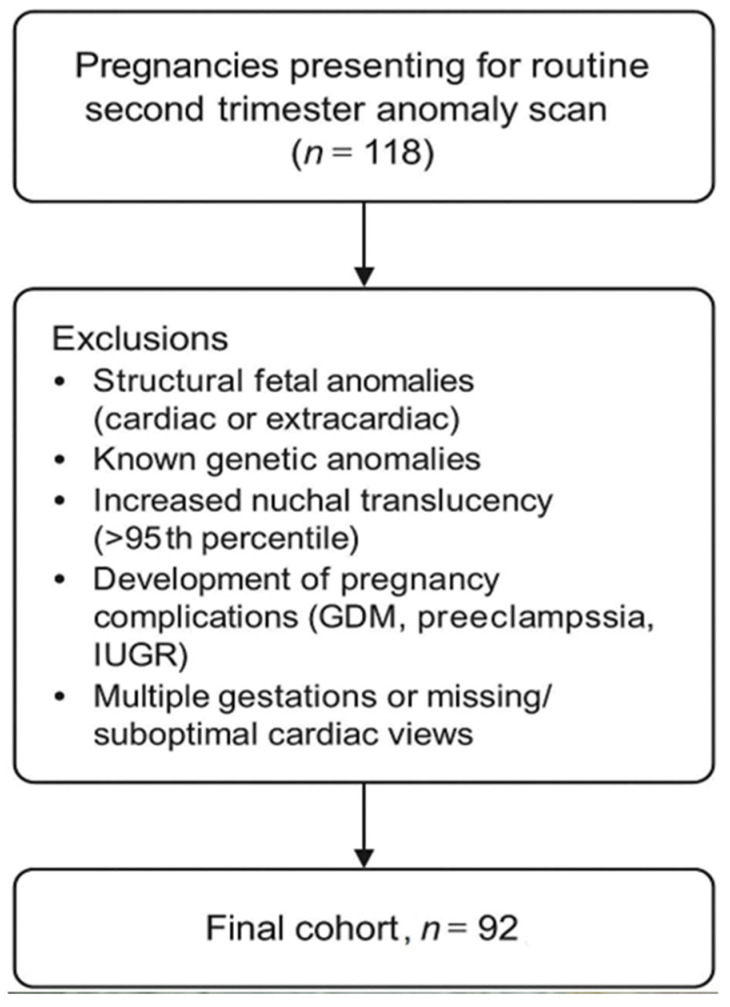
Study flow chart.

This is a prospective study conducted between October and December 2024 in an ambulatory clinic specialized in prenatal screening. A total of 118 consecutive pregnancies presenting for the second trimester anomaly scan were initially assessed for eligibility. Patients included in the study were patients that presented for the second trimester anomaly scan. The patients were selected consecutively from a list of patients; the examiner was unaware of details such as maternal age, maternal BMI (body mass index), or exact gestational age, prior to the scan. Only singleton pregnancies with normal heart anatomy were included in the cohort. Pregnancies were excluded if any structural anomaly (cardiac or extracardiac) was identified (*n* = 10), if increased first-trimester nuchal translucency (>95th percentile) had been previously documented (*n* = 3), if a known genetic diagnosis was present (*n* = 2), or if maternal–fetal complications developed later in pregnancy, including gestational diabetes, preeclampsia, or intrauterine growth restriction (*n* = 11). After applying these criteria, 92 pregnancies with normal cardiac anatomy and without maternal–fetal complications were included in the final analysis (Figure 2).

Heart measurements were performed on standard heart images recommended by the Romanian Society of Ultrasound in Obstetrics and Gynecology (SRUOG), which are the same standard planes as recommended by ISUOG (International society for Ultrasound in Obstetrics and Gynecology). In the current guidelines it is recommended to obtain the following views:

- 4 chambers
- aorta (LVOT)
- pulmonary artery (RVOT)
- 3 vessels-trachea

On the four-chamber view examiners generally save still images but also one short clip with systole and diastole, which allowed us to perform both atrial and ventricular measurements. Cardiac and thoracic dimensions were measured in the four-chamber view at end-diastole using the ellipse method to calculate the cardio-thoracic ratio. Reference points for both the heart and thorax were determined as described by Garcia-Otero. The cardiac diameters were assessed by measuring the longitudinal diameter through the ventricular septum and the transverse diameter at the level of the atrioventricular valves or at the widest points observed. Atrial measurements were performed at maximal distension during end-systole, excluding the pulmonary veins and atrioventricular valve annulus. Longitudinal and transverse diameters were determined using lines dividing each atrium into four approximately equal quadrants. Atrial areas were obtained through manual tracing. Ventricular dimensions and areas were measured in the four-chamber view at end-diastole. The basal diameters of the left and right ventricles were measured at the level of the atrioventricular valvular orifices, while midventricular diameters were assessed just below the valve leaflets. Longitudinal diameters extended from the basal diameter to the apex. Ventricular areas were obtained by manually tracing the inner ventricular border, encompassing papillary muscles and the moderator band within the ventricular cavity. The standard plane of the aorta (five-chamber view) was used to measure the aortic valve and the ascending aorta. Pulmonary valve was measured on the standard plane, with opened valve, inner edge to inner edge as described by Schneider. The three-vessels trachea view image was used to measure the ductus and aortic isthmus. The isthmus was measured proximally to the insertion of the ductus on the 3VT view as described by Pasquini. The arterial duct was measured at the level of the trachea. Most measurements were performed with the ultrasound beam orthogonal to the plane, but if the fetal position was suboptimal other angles were used as well. All measurements from the list in Table 1 were recorded in every patient that presented for second trimester anomaly scan in our unit, using the form in Appendix A. One to three measurements were performed and the one we considered most appropriate was saved and written in the form. These measurements were repeated off-line 1 to 42 days later to check for reproducibility. All examinations were performed by one examiner (A.-C.C.), ensuring internal methodological consistency and eliminating inter-observer variability for this feasibility analysis. All scans were performed using a GE Voluson E8 (BT16) ultrasound system equipped with a RAB6-D 2–7 MHz transabdominal volumetric probe manufactured by GE Healthcare, Chicago, IL, USA. The same equipment and probe settings were used throughout the study to ensure imaging consistency.

From the measurements obtained during the examinations and offline, using Excel forms with nomograms from Garcia-Otero, Schneider, and Pasquini, we calculated a number of parameters, z-scores, and centiles that are mentioned in Table 2.

Measurement analysis was assessed by comparing our results with existing nomograms obtained in studies with similar population and methodology.

All measurements were performed at the standard time for an ultrasound examination, which is 45 min in our clinic. For a better appreciation of the time needed for the measurements we analyzed the exact “screen time” for 20 cases with and 20 cases without measurements.

We compared the measurements during the scans with measurements that were performed off-line at a later date to verify reproducibility for each type of measurement.

We compared the results from the first part of the study with results from the second part (measured as deviation of z-scores from normal) to evaluate the effect of expertise on the learning curve.

Statistical analysis was performed using Microsoft Excel (Microsoft Corp., Redmond, WA, USA), together with the XLSTAT 2025.27.1.2. add-on for MS Excel (Addinsoft SARL, Paris, France). Quantitative variables were summarized as mean ± standard deviation. Normality tests (Shapiro-Wilks and Anderson–Darling) and complex statistical tests (Student *t*-test, Pearson’s r correlation coefficient) were performed using the XLSTAT addon.

For the comparison of screen-time duration between examinations with and without measurements, we computed mean differences together with 95% confidence intervals. Confidence intervals were calculated using the standard error of the mean and assuming normal distribution of the variables.

Because this project was designed as a feasibility study, the primary objective was to assess whether comprehensive cardiac morphometry could be integrated into routine second trimester scans and to describe measurement distributions and reproducibility. Therefore, no a priori sample-size calculation was performed. The sample reflects the number of consecutive eligible patients examined during the study period and provides sufficient data for descriptive and reproducibility analyses, which are appropriate for feasibility assessments.

Ethical considerations: Informed consent was obtained from all subjects involved in the study. Written informed consent was obtained from the patients to publish this paper. 

## 3. Results

The final cohort comprised ninety-two patients examined from 7 October 2024 to 20 December 2024. The average age was 29.6 years, and the average BMI was 24.2. The gestational age at examination was between 20 and 25 weeks, with an average gestational age of 22 weeks and 3 days.

Our data show that it is possible to perform a comprehensive morphometric cardiac evaluation in routine second trimester anomaly scan with acceptable accuracy and without exceeding the standard examination time. Of course, it helps that we have an above average amount of time for screening and that the measurements were made on readily available standard cardiac views.

New things this study brings are the following:

− Measurements of cardiac structures were integrated into a routine anomaly scan
− This study proves that in routine examinations, large amounts of data can be obtained from standard planes without exceeding the examination time
− This study made timing an issue of its specific objectives and proved that heart measurements do not significantly affect the examination time.

### 3.1. Feasibility

The measurements could be performed every time on the standard images and all scans were completed within the allotted time for each case. Although all measurements were successfully obtained, image quality varied between patients, and factors such as maternal BMI and fetal position occasionally required additional adjustments to acquire an adequate standard plane.

### 3.2. Measurement Analysis

**Figure 3:**
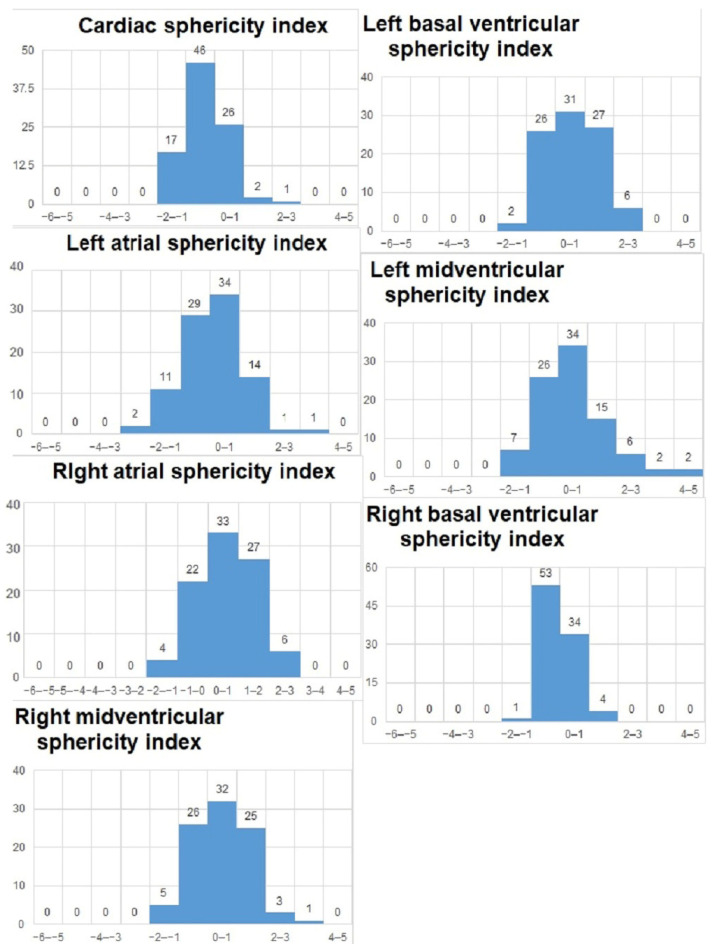
Z-scores of parameters obtained on the 4-chamber views.

**Figure 4:**
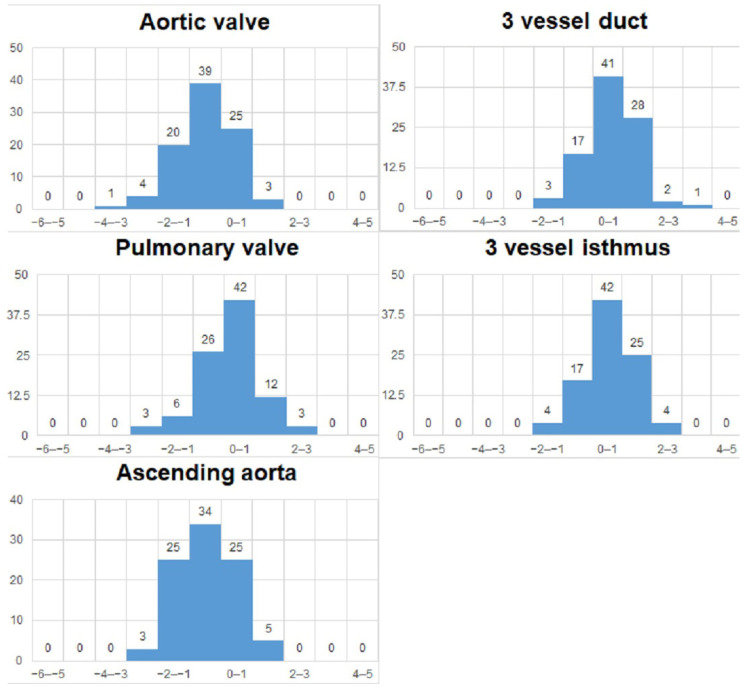
Z-scores of vessels measurements.

We analyzed the z-scores and/or centiles of the parameters from the list, as they resulted from the excel forms’ calculators and they are reported in the table below (Table 3). For almost all z-scores the values were between ±2 SD. Generally, all measured values and z-scores had a normal Gaussian distribution as tested with the Shapiro-Will and Anderson–Darling normality tests. The graphs are presented in Figure 3 and Figure 4.

### 3.3. Time

**Figure 5:**
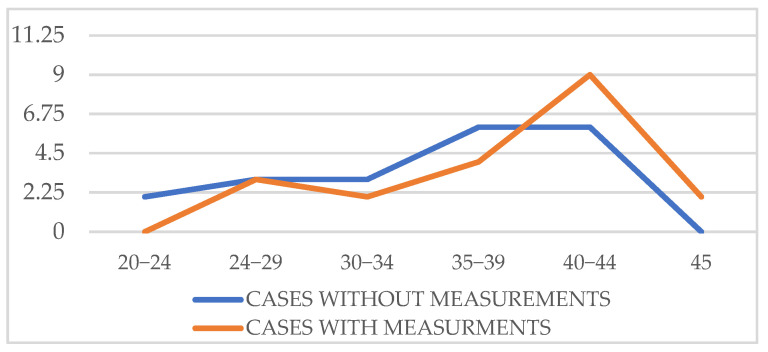
“On screen” time with and without measurements.

There was no problem in staying within our, albeit generous, time limit of 45 min. For a better appreciation of the actual “screen time”, we compared 20 cases of examinations without measurements and 20 cases of examinations with measurements. The “screen time” only included the time spent using the ultrasound machine, and not the time spent waiting for the baby to move or counseling the patient. In addition to the mean difference, we calculated 95% confidence intervals (CI) for the screen-time comparison. The mean screen time for examinations without measurements was 35.45 min (95% CI 32.9–38.0), whereas examinations with measurements required 38.75 min (95% CI 36.1–41.4). The mean difference between groups was 3.30 min (95% CI –0.21 to 6.81). As zero lies within the confidence interval, this supports the Student *t*-test finding (*p* = 0.063), indicating that the additional time required for cardiac morphometry is not statistically significant (Table 4; Figure 5).

### 3.4. Reproducibility

We compared measurements performed during examination time to measurements performed on the same images, off-line 1 to 42 days later. We compared raw measurements, z-scores ± centiles.

**Figure 6:**
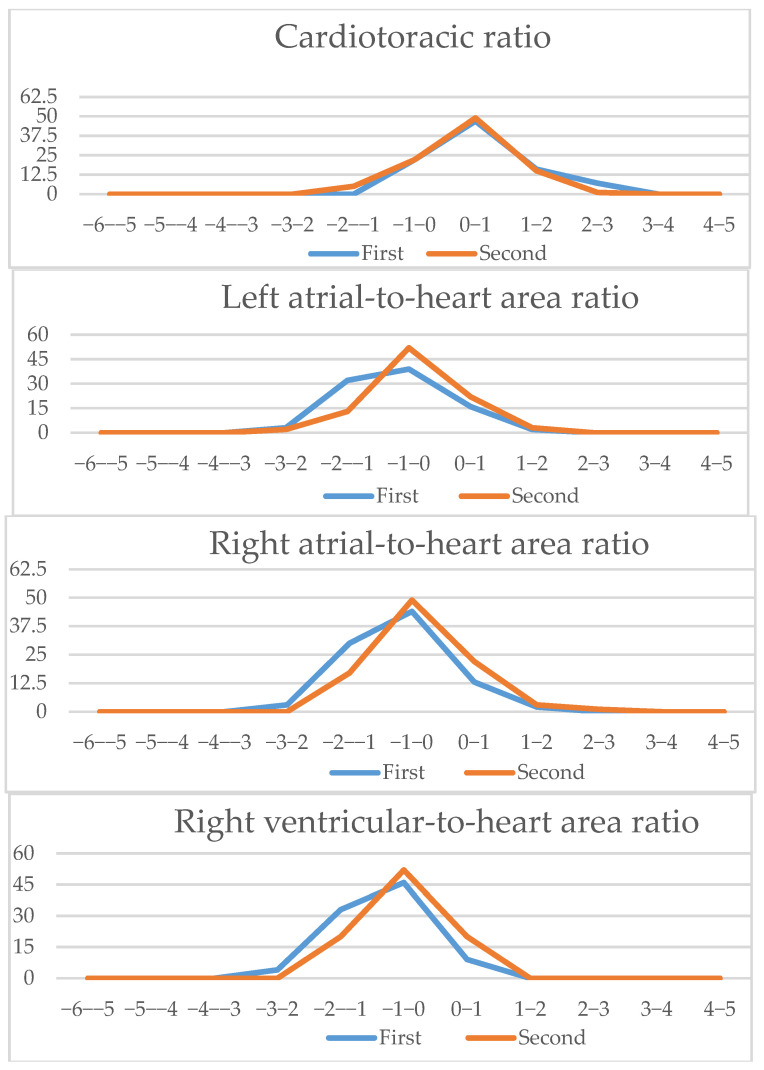

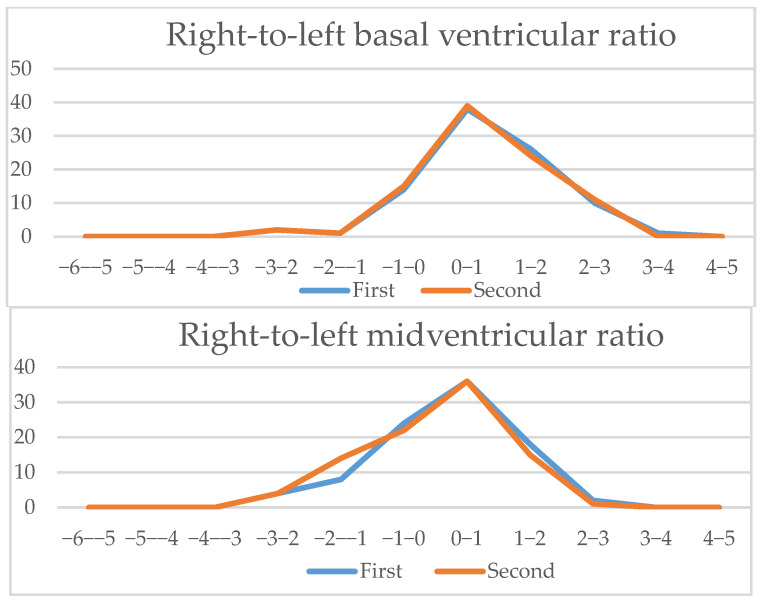
Four-chamber view parameters (1): Z-scores comparison of live-scan (first) and off-line (second) measurements (CTAR, left atria/heart ratio, right atria/heart ratio, right ventricle/heart ratio, right/left basal ventricular ratio, right/left midventricular ratio).

**Figure 7:**
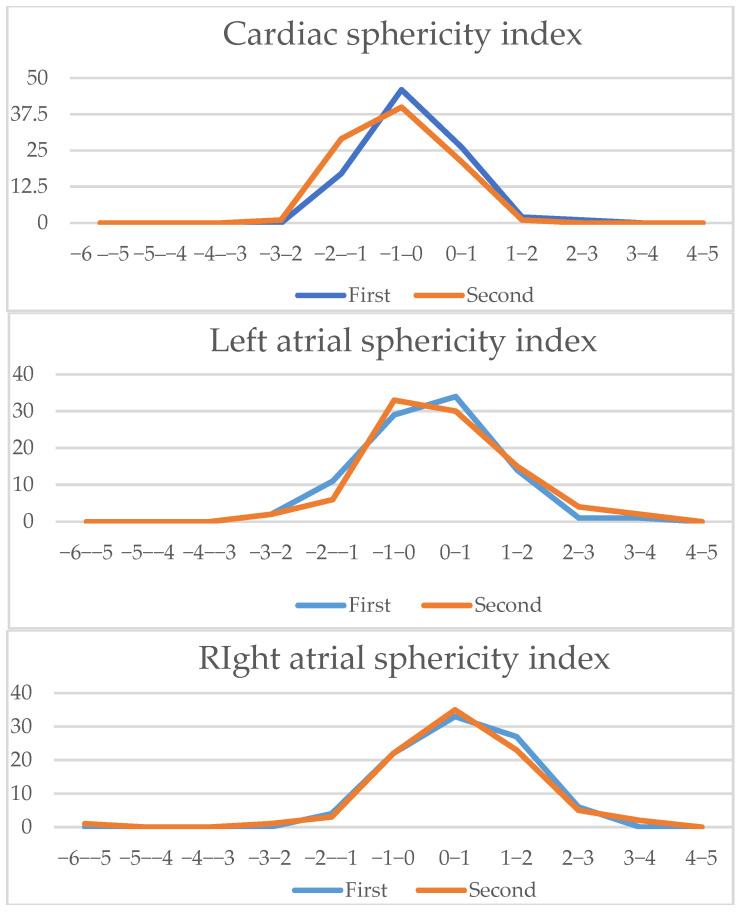

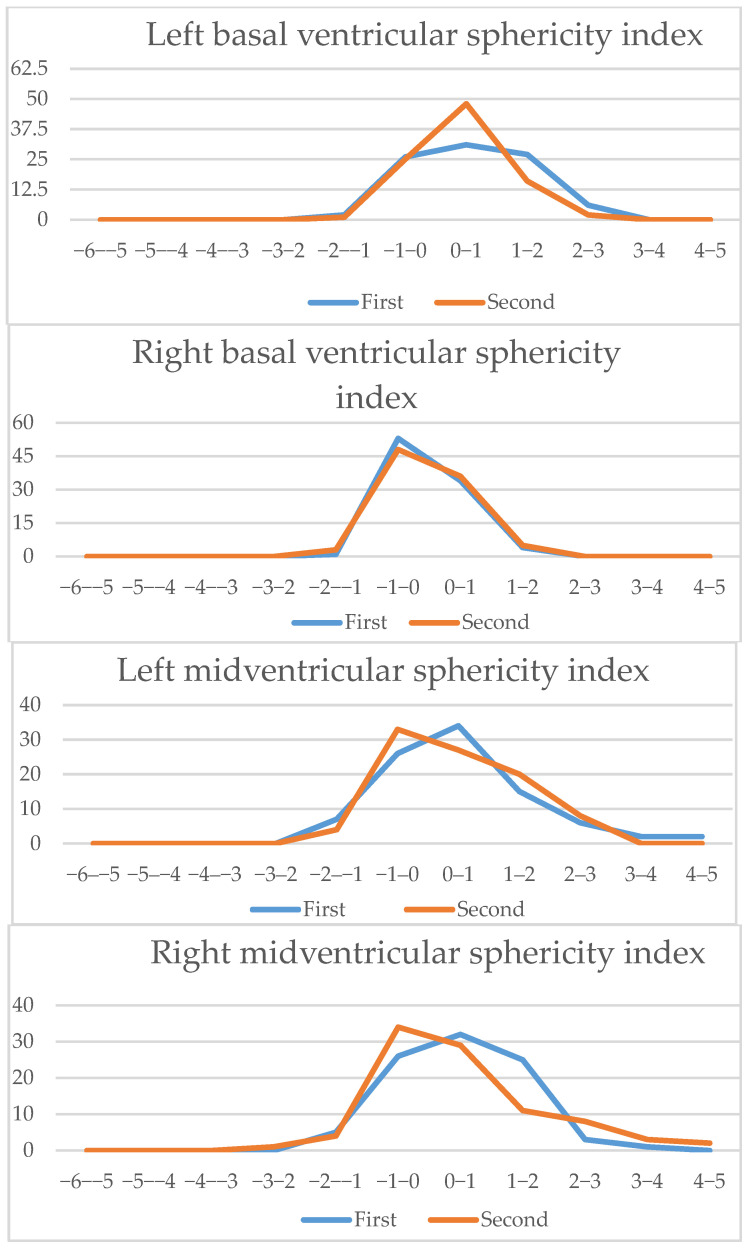
Four-chamber view parameters (2): Z-scores comparison of live-scan (first) and off-line (second) measurements (cardiac sphericity index, left atria sphericity index, right atria sphericity index, left basal ventricular sphericity index, right basal ventricular sphericity index, left midventricular sphericity index, right midventricular sphericity index).

**Figure 8:**
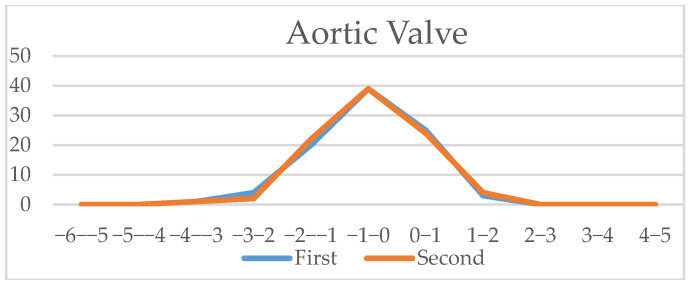

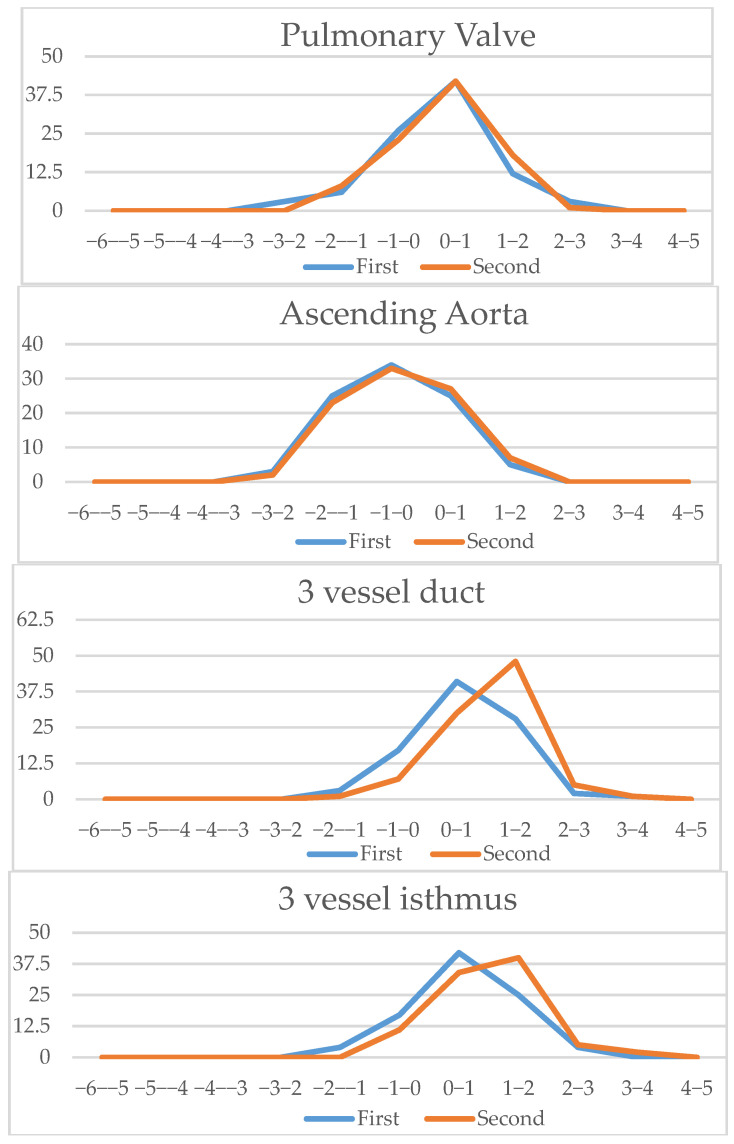
Vessels (aortic valve, pulmonary valve, ascending aorta, three vessel duct and aortic isthmus): Z-scores comparison of live-scan (first) and off-line measurements (second).

Since all measured values and z-scores had a normal Gaussian distribution, we could use the parametric Student *t*-test for paired data. We noticed that for certain parameters, there were significant differences when assessing if the measurement allowed for zero error. We also calculated the Pearson correlation coefficient to assess the similarity between the intra-observer measurements. The measurements’ results showed an excellent correlation coefficient for cardiac area, thoracic area, longitudinal cardiac diameter, transverse cardiac diameter, right ventricle area, left ventricle area, left ventricle longitudinal diameter, right atrial area, left atrial area, aortic valve and pulmonary valve, a good correlation coefficient for ascending aorta, three vessel isthmus, right ventricular longitudinal diameter, right ventricular basal diameter, left ventricular basal diameter, right ventricle midtransverse diameter, left ventricle midtransverse diameter, right atrial longitudinal diameter, left atrial longitudinal diameter, left atrial transverse diameter, and a fair correlation was noticed for right atrial transverse diameter and three vessel duct measurement. A detailed table of all measurements, z-scores, and centiles correlation coefficients is available in Appendix B; in Figure 6, Figure 7 and Figure 8 a graphic comparison of z-scores is available.

### 3.5. Learning Curve

We compared the first 46 sets of measurements z-scores with the last 46 to check if they are significantly closer to 0 (hence, theoretically better in the second half of the study) using the Student *t*-test (Table 5). Regarding whether there is an improvement of measurement proficiency (measured by z-scores deviation) throughout the study (learning curve), we noticed this disposition with statistical significance (*p* < 0.05) only for a few parameters; we also noticed that for some parameters measurements had a smaller deviation from zero, but without statistical significance. The parameters that showed statistically significant measurement improvement were left atrial-to-heart area ratio, right atrial-to-heart area ratio, right ventricular-to-heart area ratio, and pulmonary valve.

## 4. Discussion

This study targeted mainly the feasibility of cardiac morphometry in the routine anomaly scan. Other studies were designed to create nomograms for singleton pregnancies (Schneider [11], Garcia-Otero [6]) or to offer nomograms to help with a specific diagnosis—like the study of Pasquini [12] for the coarctation of the aorta. The study of Frandsen [7] assessed the cardiac morphometry in babies with IUGR. In other studies measurements are performed differently, like in the study of Vigneswaran [13] where the aortic and pulmonary valve measurements are made with closed valves. Retrospectively, we consider that this type of measurement might be more reproducible but, in our study, we followed the measurements from Schneider et al. [11] that we chose as part of our protocol at the beginning of the study. We acknowledge that the novelty of the study is moderate, as cardiac morphometry and its reference ranges have been extensively investigated in previous work. Nevertheless, our study provides new practical evidence that these measurements can be feasibly integrated into routine anomaly scans, using standard imaging planes, without significantly increasing examination time. This perspective is clinically relevant, especially in general obstetric practice where detailed fetal echocardiography may not always be available.

An additional key limitation is the single-center, single-operator design. All examinations and all offline measurements were performed by the same experienced examiner, who ensured internal consistency and minimized measurement variability within the study. However, this approach inherently limits the generalizability of our findings, as it does not account for inter-observer variability or differences in operator experience. Operator dependence is intrinsic to ultrasound-based techniques, and further multicenter studies including examiners with varying levels of expertise are needed to assess whether these measurements can be reliably reproduced in broader clinical settings.

A limitation of the study is that our final cohort represents an idealized, highly selected population rather than a typical low-risk screening population. Excluding all structural anomalies and maternal–fetal complications may limit the generalizability of our findings to broader obstetric settings. The cohort consisted of pregnancies from a relatively homogeneous regional population, with generally favorable acoustic conditions and low-to-moderate maternal BMI. This likely facilitated cardiac image acquisition and contributed to the short scan times observed. Therefore, the external applicability of our results to populations with higher BMI, different body habitus, or more challenging acoustic windows may be limited. Future studies should validate the feasibility of routine cardiac morphometry in more diverse populations and across a broader range of maternal and fetal conditions.

In this study, we intentionally chose a “single-best-measurement” strategy rather than averaging multiple repeated measurements. This decision was driven by the primary aim of evaluating whether cardiac morphometry can be integrated into routine second-trimester scans without extending examination time. Averaging repeated measurements, although methodologically ideal, would not reflect real-life workflow in a typical anomaly scan, where time constraints are substantial and clinicians commonly retain the clearest single measurement. However, we recognize that this approach influences the interpretation of reproducibility indices, as intra-observer comparisons were based on the “best of a series” rather than on averaged values. This represents a meaningful limitation, and future studies should incorporate standardized repeated measurements to provide more robust repeatability estimates.

Some of the not so good results (according to z-scores) or lesser reproducibility rates were the result of inappropriate measurements, partly due to inexperience in cardiac morphometry but also, as we retrospectively noticed, those tended to be at the end of the program. Other problems associated with inaccurate measurements—noticed after calculating the z-scores—was a high maternal BMI. The positive side of this research study is that it proves that cardiac measurements can be part of the routine scans, when necessary, without exceeding the standard examination time and with minimal training. It is fair to mention that the timing results that are compared are obtained in the second part of the study, and the examiner was aware of being timed.

Another important limitation of our study is its single-center design and the fact that all examinations were performed by a single experienced operator. While this ensured internal consistency and reduced inter-observer variability, it also limited the generalizability of our findings. In addition, the study population consisted exclusively of uncomplicated singleton pregnancies with optimal cardiac views, representing an idealized cohort rather than a true low-risk screening population. Therefore, although our results suggest that cardiac morphometry can be integrated into routine anomaly scans without prolonging examination time, these findings should be interpreted with caution and validated in broader, multi-center settings with examiners of varying levels of experience.

Although measurements were feasible in all cases, this does not imply that image quality was uniform across the cohort. In several examinations, suboptimal fetal position or increased maternal BMI temporarily reduced image clarity, requiring additional time or technical adjustments. Therefore, the statement that measurements “could be done every time” reflects feasibility rather than uniform ease of acquisition.

Future studies should focus on potential benefits of using a smaller set of measurements, perhaps as screening tools: for example, to test if there is a cut-off in the z-scores for isthmus measurement or right-to-left midventricular ratio to define a population at high risk for coarctation of the aorta?

Reproducibility assessment in our study relied on Pearson’s correlation coefficient, which measures linear association but not absolute agreement. We acknowledge that intraclass correlation coefficients (ICC) and Bland–Altman analyses represent the gold standard for repeatability studies. These methods were not included because the primary aim of this project was feasibility rather than full measurement validation, and the study design involved single repeated measurements rather than repeated independent acquisitions suited for ICC modeling. Future studies, ideally multicentric and involving multiple operators, should incorporate ICC and Bland–Altman plots to provide a more robust assessment of measurement agreement.

Another limitation concerns the statistical handling of the learning-curve analysis. Because multiple parameters were compared between the first and second half of the study, the risk of type I error is increased. No correction for multiple comparisons (such as Bonferroni or Holm adjustment) was applied, as the analysis was exploratory and aimed only to identify general trends rather than to establish definitive statistical thresholds. Consequently, the few parameters reaching *p* < 0.05 should be interpreted with caution, as they may reflect chance findings rather than true improvements in operator performance.

A different approach that we would consider for a more practical endeavor would be to discard the use of excel sheets’ calculators (although they provide more parameters and are more appropriate for constructing a data base) and use Fetal Barcelona Calculators app [16], which, later in the project, we realized was much more user friendly and did not require access to a laptop, while offering enough information in a clinical setting.

Although automated and AI-assisted tools are increasingly integrated into modern ultrasound platforms, manual measurements remain essential in current clinical practice. Automated algorithms still require high-quality acoustic windows, consistent frame selection, and operator oversight, and inaccuracies may occur in suboptimal conditions. Therefore, a solid understanding of manual morphometric assessment remains important to ensure that automated outputs are correctly interpreted and verified. Our findings support the continued relevance of manual measurements by showing that, when performed on standard planes during routine scans, they can be obtained within the allotted examination time and with acceptable reproducibility. As automation evolves, maintaining proficiency in manual techniques ensures that clinicians can both validate automated results and perform reliable measurements when automated tools are unavailable or inconclusive.

In recent years, automated and semi-automated tools for fetal biometry have gained increasing relevance, particularly for structures that are static or less affected by fetal motion. For example, automated neurosonography platforms such as Samsung 5D CNS+ [19] and GE SonoCNS [20] have demonstrated that standardized plane acquisition and algorithm-based measurements can significantly reduce operator dependence and improve reproducibility. Although the fetal heart represents a greater technical challenge due to its continuous movement and the need for cardiac-cycle synchronization, several exploratory systems are now emerging, suggesting that cardiac morphometry may also become partially automated in the near future. Nonetheless, current automated solutions remain limited by image quality, acoustic windows, motion artifacts, and the need for precise frame selection, meaning that manual assessment remains necessary in routine clinical practice. Our findings support this perspective: even with manual measurements performed on standard planes, reproducibility was acceptable and examination time was not significantly increased. Thus, while automation is an important and promising direction, real-world integration will require validation across diverse populations, equipment, and operator skill levels.

## 5. Conclusions

Our research proves that measurements of cardiac structures can be performed in a routine setting on standard planes and they do not significantly affect the examination time (the set time for anomaly scan in our clinic was never exceeded). Z-scores for measured parameters were similar to other studies with the same type of population and methodology and our measurements, performed in the routine scan, were reasonably reproducible. Measurements performed similarly throughout the research. Impact of acquired expertise was only significant for some parameters (left atrial-to-heart area ratio, right atrial-to-heart area ratio, right ventricular-to-heart area ratio, and pulmonary valve).

## Figures and Tables

**Table 1:** The measurement list used in our study.

4CV and Thoracic End-Diastolic Measurements
Cardiac Area
Thoracic Area
Longitudinal Cardiac Diameter
Transverse Cardiac Diameter
Ventricular End-Diastolic Measurements
RV Area
LV Area
RV Longitudinal Diameter
LV Longitudinal Diameter
RV Basal Diameter
LV Basal Diameter
RV Midtransverse Diameter
LV Midtransverse Diameter
Atrial End-Systolic Measurements
RA Area
LA Area
RA Longitudinal Diameter
LA Longitudinal Diameter
RA Transverse Diameters
LA Transverse Diameters
Vessels
Aortic Valve
Pulmonary Valve
Ascending Aorta
3 vessel duct
3 vessel isthmus

**Table 2:** Parameters obtained from our measurements (measurements, z-scores +/− centiles).

4-Chambers View—Measurements, Z-Scores, and Centiles (Nomograms from Garcia-Otero [6])
Cardiothoracic ratio
Left atrial-to-heart area ratio
Right atrial-to-heart area ratio
Right ventricular-to-heart area ratio
Cardiac sphericity index
Left atrial sphericity index
Right atrial sphericity index
Left basal ventricular sphericity index
Left midventricular sphericity index
Right basal sphericity index
Right midventricular sphericity index
Right-to-left basal ventricular ratio
Right-to-left midventricular ratio
Vessels—Z scores (nomograms from Schneider [11] and Pasquini [12])
Aortic Valve
Pulmonary Valve
Ascending Aorta
3 vessel duct
3 vessel isthmus

**Table 3:** Results of z-scores and centiles for parameters obtained from our measurements.

4-chamber view—Z-scores (nomograms from Garcia-Otero [6])	
Cardiothoracic ratio	+0.33 (−1.79–0.94)
Left atrial-to-heart area ratio	−0.37 (−2.51–+1.21)
Right atrial-to-heart area ratio	−0.40 (−1.72–+2.13).
Right ventricular-to-heart area ratio	−0.49 (−1.98–+0.98).
Cardiac sphericity index	−0.56 (−2.04–+1.45)
Left atrial sphericity index	+0.23 (−2.69–+3.1)
Right atrial sphericity index	+0.47 (−6.35–+3.3)
Left basal ventricular sphericity index	+0.40 (−1.40–2.07)
Left midventricular sphericity index	0.39 (−1.56–+2.51)
Right basal sphericity index	−0.04 (−1.44–+1.77)
Right midventricular sphericity index	+0.54 (−2.08–+4.18)
Right-to-left basal ventricular ratio	+0.73 (−2.48–+2.99)
Right-to-left midventricular ratio	+0.04 (−2.28–+2.39)
4-chamber view—centiles (nomograms from Garcia-Otero [6])	
Cardiothoracic ratio	66% (22.74–99.64%)
Left atrial-to-heart area ratio	27.5% (0.35–85.27%)
Right atrial-to-heart area ratio	26.64% (0.72–92.4%)
Right ventricular-to-heart area ratio	23.38% (0.14–70.76%)
Cardiac sphericity index	38.2% (2.8–98.36%)
Left atrial sphericity index	52.55% (0.49–99.94%)
Right atrial sphericity index	65.09% (3.12%99.65%)
Left basal ventricular sphericity index	65.2% (11.03–99.46%)
Left midventricular sphericity index	60.65 (2.28–100%)
Right basal sphericity index	47.86% (7.46–96.18%)
Right midventricular sphericity index	61.21 (2.45–99.89%)
Right-to-left basal ventricular ratio	71% (0.42–99.8%)
Right-to-left midventricular ratio	54.55% (0.53–98,59%)
Vessels—Z scores (nomograms from Schneider [11] and Pasquini [12])	
Aortic valve	−0.48 (−3.37–+1.55)
Pulmonary valve	0.11 (−2.25–+2.58)
Ascending aorta	−0.55 (−2.66–+1.29)
3 vessel duct	+0.63 (−1.89–+2.16)
3 vessel isthmus	+0.49 (−1.55–+2.7)

**Table 4:** Timing of actual “screen time” for cases with and without heart measurements.

	Cases Without Measurements	Cases With Measurments
1	32	37
2	29	29
3	40	40
4	23	44
5	36	44
6	38	42
7	43	36
8	38	45
9	41	33
10	42	41
11	25	29
12	34	28
13	37	42
14	41	39
15	33	41
16	28	32
17	24	42
18	39	43
19	38	45
20	40	35

**Table 5:** Comparing the first vs. the last sets of measurements—statistical analysis.

Variable	Mean 1–46	StDev 1–46	Mean 47–92	StDev 47–92	*p* Test Student	Z1–46 > Z47–92
Cardiothoracic ratio	0.67	0.78	0.47	0.83	0.2332	TRUE
Left atrial-to-heart area ratio	−1.03	0.65	−0.46	0.75	0.0002	TRUE
Right atrial-to-heart area ratio	−1.16	0.58	−0.37	0.64	0.0000	TRUE
Right ventricular-to-heart area ratio	−1.10	0.70	−0.65	0.58	0.0013	TRUE
Cardiac sphericity index	−0.34	0.87	−0.37	0.69	0.8637	FALSE
Left atrial sphericity index	−0.26	0.91	0.46	1.00	0.0005	FALSE
Right atrial sphericity index	0.29	1.07	0.79	0.75	0.0110	FALSE
Left basal ventricular sphericity index	0.67	0.86	0.42	0.92	0.1968	TRUE
Left midventricular sphericity index	0.59	1.17	0.44	1.36	0.5743	TRUE
Right basal ventricular sphericity index	0.07	0.61	−0.16	0.56	0.0656	FALSE
Right midventricular sphericity index	0.32	1.07	0.48	0.91	0.4457	FALSE
Right-to-left basal ventricular ratio	0.58	1.06	0.95	0.93	0.0750	FALSE
Right-to-left midventricular ratio	0.18	1.10	0.07	0.92	0.6158	TRUE
Aortic valve	−0.54	0.86	−0.43	0.93	0.5526	TRUE
Pulmonary valve	0.33	0.98	−0.10	0.89	0.0310	TRUE
Ascending aorta	−0.71	0.85	−0.37	0.92	0.0661	TRUE
3 vessel duct	0.54	0.92	0.77	0.77	0.2068	FALSE
3 vessel isthmus	0.39	0.86	0.64	0.85	0.1517	FALSE

## Data Availability

The data presented in this study are available on request from the corresponding author due to privacy and ethical restrictions related to patient confidentiality and informed consent.

## Appendix A

**Table A1:** Measurement protocol form.

Patient Data		
Name		
Gestational age (MA)		
Gestational age (sonographic)		
Date measurement 1		
Date measurement 2		
	
**Measurements**	Measurement 1	Measurement 2
4CV and Thoracic End-Diastolic Measurements		
Cardiac Area		
Thoracic Area		
Longitudinal Cardiac Diameter		
Transverse Cardiac Diameter		
Ventricular End-Diastolic Measurements		
RV Area		
LV Area		
RV Longitudinal Diameter		
LV Longitudinal Diameter		
RV Basal Diameter		
LV Basal Diameter		
RV Midtransverse Diameter		
LV Midtransverse Diameter		
Atrial End-Systolic Measurements		
RA Area		
LA Area		
RA Longitudinal Diameter		
LA Longitudinal Diameter		
RA Transverse Diameters		
LA Transverse Diameters		
Vessels		
Aortic Valve		
Pulmonary Valve		
Ascending Aorta		
3 vessel duct		
3 vessel isthmus		

## Appendix B

**Table A2:** Pearson’s correlation coefficient of studied parameters.

Variable	Correl Coef		
Cardiac Area	0.862		
Thoracic Area	0.860		
Longitudinal Cardiac Diameter	0.799		
Transverse Cardiac Diameter	0.867		
Ventricular End-Diastolic Measurements			Correl coef
RV Area	0.859	>0.75	Excellent
LV Area	0.838	0.6–0.75	Good
RV Longitudinal Diameter	0.749	0.4–0.6	Fair
LV Longitudinal Diameter	0.796	<0.4	Poor
RV Basal Diameter	0.676		
LV Basal Diameter	0.720		
RV Midtransverse Diameter	0.664		
LV Midtransverse Diameter	0.682		
Atrial End-Systolic Measurements			
RA Area	0.852		
LA Area	0.773		
RA Longitudinal Diameter	0.666		
LA Longitudinal Diameter	0.698		
RA Transverse Diameters	0.542		
LA Transverse Diameters	0.728		
**Value**			
Cardiothoracic ratio	0.516		
Left atrial-to-heart area ratio	0.381		
Right atrial-to-heart area ratio	0.651		
Left ventricular-to-heart area ratio	0.497		
Right ventricular-to-heart area ratio	0.495		
Cardiac sphericity index	0.543		
Left atrial sphericity index	0.653		
Right atrial sphericity index	0.162		
Left basal ventricular sphericity index	0.617		
Left midventricular sphericity index	0.609		
Right basal ventricular sphericity index	0.490		
Right midventricular sphericity index	0.581		
Right-to-left basal ventricular ratio	0.369		
Right-to-left midventricular ratio	0.463		
**Z-scores**			
Cardiothoracic ratio	0.510		
Left atrial-to-heart area ratio	0.384		
Right atrial-to-heart area ratio	0.639		
Left ventricular-to-heart area ratio			
Right ventricular-to-heart area ratio	0.512		
Cardiac sphericity index	0.539		
Left atrial sphericity index	0.613		
Right atrial sphericity index	0.380		
Left basal ventricular sphericity index	0.617		
Left midventricular sphericity index	0.610		
Right basal ventricular sphericity index	0.523		
Right midventricular sphericity index	0.579		
Right-to-left basal ventricular ratio	0.399		
Right-to-left midventricular ratio	0.463		
**Centiles**			
Cardiothoracic ratio	0.496		
Left atrial-to-heart area ratio	0.349		
Right atrial-to-heart area ratio	0.631		
Left ventricular-to-heart area ratio			
Right ventricular-to-heart area ratio	0.466		
Cardiac sphericity index	0.524		
Left atrial sphericity index	0.591		
Right atrial sphericity index	0.361		
Left basal ventricular sphericity index	0.590		
Left midventricular sphericity index	0.574		
Right basal ventricular sphericity index	0.386		
Right midventricular sphericity index	0.668		
Right-to-left basal ventricular ratio	0.403		
Right-to-left midventricular ratio	0.471		
**Vessels Value**			
Aortic Valve	0.783		
Pulmonary Valve	0.829		
Ascending Aorta	0.669		
3 vessel duct	0.583		
3 vessel isthmus	0.731		
**Z-scores**			
Aortic Valve	0.660		
Pulmonary Valve	0.726		
Ascending Aorta	0.787		
3 vessel duct	0.523		
3 vessel isthmus	0.596		
